# Associations of HDL Subspecies Defined by ApoC3 with Non-Alcoholic Fatty Liver Disease: The Multi-Ethnic Study of Atherosclerosis

**DOI:** 10.3390/jcm9113522

**Published:** 2020-10-31

**Authors:** Jakub Morze, Manja Koch, Sarah A. Aroner, Matthew Budoff, Robyn L. McClelland, Kenneth J. Mukamal, Majken K. Jensen

**Affiliations:** 1 Department of Cardiology and Internal Medicine, University of Warmia and Mazury, 11-041 Olsztyn, Poland; jakub.morze@uwm.edu.pl; 2 Department of Nutrition, Harvard T.H. Chan School of Public Health, Boston, MA 02115, USA; mkoch@hsph.harvard.edu (M.K.); kmukamal@bidmc.harvard.edu (K.J.M.); 3 Department of Psychiatry, Massachusetts General Hospital, Harvard Medical School, Boston, MA 02115, USA; saroner@mgh.harvard.edu; 4 Harbor-UCLA Medical Center, Los Angeles Biomedical Research Institute, Los Angeles, CA 90095, USA; mbudoff@labiomed.org; 5 Department of Biostatistics, University of Washington, Seattle, WA 98195-7232, USA; rmcclell@uw.edu; 6 Department of Medicine, Beth Israel Deaconess Medical Center, Boston, MA 02115, USA; 7 Department of Public Health, Section of Epidemiology, University of Copenhagen, DK-1353 Copenhagen, Denmark

**Keywords:** high-density lipoprotein, apolipoprotein C3, liver fat, NAFLD

## Abstract

Previously, we reported that inverse associations of high-density lipoprotein (HDL) with cardiovascular disease and diabetes were only observed for HDL that lacked the pro-inflammatory protein apolipoprotein C3 (apoC3). To provide further insight into the cardiometabolic properties of HDL subspecies defined by the presence or absence of apoC3, we aimed to examine these subspecies with liver fat content and non-alcoholic fatty liver disease (NAFLD). We investigated cross-sectional associations between ELISA-measured plasma levels of apoA1 in HDL that contained or lacked apoC3 and computed tomography-determined liver fat content and NAFLD (<51 HU) at baseline (2000–2002) among 5007 participants in the Multi-Ethnic Study of Atherosclerosis (MESA) without heavy alcohol consumption (>14 drinks/week in men and >7 drinks/week in women). In multivariable-adjusted regression models, apoA1 in HDL that contained or lacked apoC3 was differentially associated with liver fat content (*P*_heterogeneity_ = 0.048). While apoA1 in HDL that lacked apoC3 was inversely associated with liver fat content (*P*_trend_ < 0.0001), apoA1 in HDL that contained apoC3 was not statistically significantly associated with liver fat content (*P*_trend_ = 0.57). Higher apoA1 in HDL that lacked apoC3 was related to a lower prevalence of NAFLD (OR per SD: 0.80; 95% CI: 0.72, 0.89), whereas no association was found for apoA1 in HDL that contained apoC3 (OR per SD: 0.95; 95% CI: 0.85, 1.05; *P*_heterogeneity_ = 0.09). Higher apoA1 in HDL that lacked apoC3 was associated with less liver fat content and a lower prevalence of NAFLD. This finding extends the inverse association of HDL lacking apoC3 from cardiovascular disease to NAFLD. Lack of biopsy-proven hepatic steatosis and fibrosis data requires the replication of our study in further studies.

## 1. Introduction

Non-alcoholic fatty liver disease (NAFLD) is characterized by excessive hepatic fat accumulation in the absence of heavy alcohol intake. NAFLD remains one of the major causes of chronic liver disease, increasing risk of liver cirrhosis and hepatocellular cancer [1]. The prevalence of NAFLD is estimated to be 25.2% worldwide, with a higher prevalence in the Middle East and South America and a lower prevalence in Africa [2].

NAFLD frequently occurs in conjunction with obesity, insulin resistance, and dyslipidemia, conditions encompassed in the definition of the metabolic syndrome (MetS). Patients with MetS show increased catabolism of high-density lipoprotein (HDL), leading to decreased HDL cholesterol levels. Lower HDL cholesterol is commonly present in NAFLD. It has become increasingly clear that HDL comprises subclasses that are heterogeneous in size, composition, and metabolic function [3]. Accumulating evidence suggests that the physiological roles of HDL depend on its protein cargo. In addition to apoA1, the main structural protein component of HDL, over 100 other proteins have been identified as HDL-associated protein [4]. One of the most abundant proteins in HDL, beyond apoA1, is apolipoprotein C3 (apoC3), a pro-inflammatory glycoprotein primarily synthesized in the liver [5]. Previous research has suggested that polymorphisms in the *APOC3* gene encoding the apoC3 protein might be responsible for genetic susceptibility to NAFLD [6]. A recent randomized trial with an antisense inhibitor of apoC3 synthesis showed a significant decrease in fasting triglycerides, highlighting apoC3 as upstream of triglycerides [7]. Therefore, apoC3 has the potential to regulate liver fat accumulation by influencing circulating triglycerides.

About half of circulating apoC3 protein is present in HDL, and HDL that contains apoC3 represents more than 6% of total HDL [8]. Previously, we showed that higher apoA1 in HDL that contained apoC3 is associated with a higher risk of type 2 diabetes and coronary heart disease, whereas higher apoA1 in HDL that lacked apoC3 was associated with a lower risk of type 2 diabetes and coronary heart disease [9,10]. Based upon these previous findings on diabetes and coronary heart disease risk, two conditions strongly linked to NAFLD [9,11], we examined the association of apoA1 in HDL that contained or lacked apoC3 with liver fat content and NAFLD in the Multi-Ethnic Study of Atherosclerosis (MESA).

## 2. Materials and Methods

### 2.1. Study Population and Design

We conducted a cross-sectional study at baseline (2000 to 2002) of participants enrolled in MESA. The design and methods of MESA have been described previously [12]. In brief, MESA is a population-based prospective cohort study that recruited 6814 community-dwelling participants aged 45–84 years at baseline. Participants free of clinical cardiovascular disease were recruited from six academic centers in the United States.

**Figure 1:**
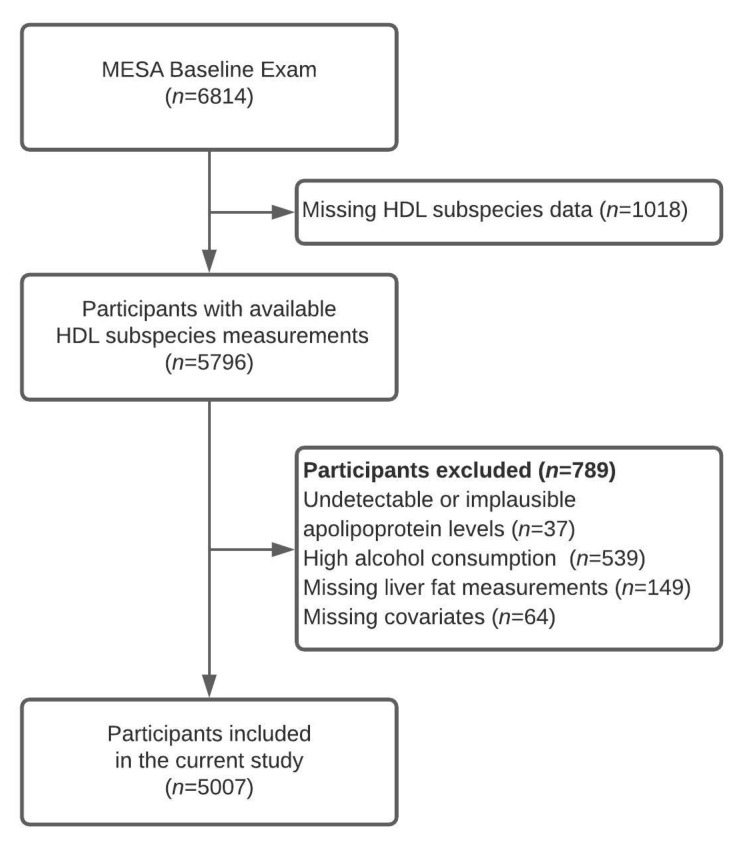
Flowchart presenting study sample selection process. MESA—Multi-Ethnic Study of Atherosclerosis.

In the present analysis (Figure 1), of the 6814 recruited participants we included 5796 of those who had HDL subspecies measured at baseline. We excluded 37 participants with undetectable or implausibly low apolipoprotein values, 539 participants with high alcohol consumption (>14 drinks per week for men and >7 drinks per week for women [13]), 149 participants without liver fat content measurements, and 64 participants missing covariates, leaving 5007 participants in the final analysis. At baseline, participants provided data on demographics, lifestyle factors, and medical history using standardized questionnaires [12]. Trained personnel measured height and weight. Study participants reported physical activity level by the time spent on moderate-to-vigorous activities. Institutional Review Boards of each study site (Baltimore, MD; Chicago, IL; Forsyth County, NC; Los Angeles, CA; New York, NY; and St. Paul, MN) approved the study protocol, and all participants provided written, informed consent.

### 2.2. Assessment of Apolipoprotein Levels

The lipid lab at the Harvard T.H. Chan School of Public Health measured apolipoproteins in blood plasma samples collected at baseline from 5796 participants using a sandwich ELISA (Academy Biomedical Company, Houston, TX, USA). Trained personnel collected samples from participants after overnight fasting and then stored them at −80 °C until measurement in 2014. A detailed protocol of HDL subspecies quantification has been published elsewhere [11]. We used the concentration of apoA1, the main structural protein of HDL, to quantify HDL levels. First, we measured the concentrations of apoC3 and apoA1 in whole plasma. After the whole plasma was fractionated by immunoaffinity separation into lipoproteins containing or lacking apoC3, we assessed the concentration of apoA1 in HDL containing apoC3 and apoA1 in HDL lacking apoC3.

Laboratory personnel performed all apolipoprotein measurements in duplicate and were unaware of participant NAFLD status. Average within-run coefficients of variation were 4% for total apoC3, 5% for apoA1 in HDL that lacked apoC3, and 8% for apoA1 in HDL that contained apoC3.

### 2.3. Assessment of Liver Fat Content and Non-Alcoholic Fatty Liver Disease

During a single session, each participant underwent two consecutive cardiac non-contrast-enhanced computer tomography scans covering the liver and spleen, as reported previously [14]. Two readers independently assessed the scans. Liver fat content was approximated by hepatic signal attenuation in Hounsfield units (HU). HU values were measured in three regions of interest: two regions in the right liver lobe anteroposteriorly and one in the left lobe, and then averaged. We selected liver fat content as our primary continuous outcome to maximize statistical power. Presence of NAFLD was classified using a cut-off point for hepatic signal attenuation <51 HU.

### 2.4. Statistical Analysis

We recalibrated concentrations of apolipoproteins and HDL subspecies according to methods developed by Rosner et al. to account for batch effects [15]. In brief, we regressed log-transformed apolipoprotein levels for batch, age, sex, race, study site, smoking status, alcohol intake, education, and body mass index (BMI). Within each batch, biomarkers were recalibrated by adding the resulting value for the batch indicator minus the average of the combined batch coefficient [15]. We evaluated the baseline characteristics of men and women separately. We assessed the correlation of untransformed apoC3 and triglycerides using Spearman rank correlation coefficients.

We used general linear regression models to evaluate associations between untransformed plasma apolipoproteins levels (total apoC3, total apoA1, ApoA1 in HDL that contained apoC3, and apoA1 in HDL that lacked apoC3) and liver fat content at baseline. We used logistic regression models to assess the relationship between apolipoproteins and NAFLD prevalence. The concentration of apolipoproteins was modeled continuously and as quintiles.

In Model 1, we adjusted for age, race/ethnicity (White, African-American, Chinese-American, and Hispanic), sex, and study site. Model 2 was further adjusted for income category (<$25,000, $25,000–$49,999, $50,000–$74,999, and ≥$75,000 per year), cigarette smoking status (current, former, and never), alcohol consumption (current, former, and never) and alcohol amount (g/day), time spent on moderate-to-vigorous physical activity (MET-hr/wk), and BMI (liver-fat-adjusted, kg/m^2^). All analyses of apoA1 in HDL that contained or lacked apoC3 were mutually adjusted for the complementary HDL subspecies. Wald tests were used to assess slope heterogeneity of apoA1 in HDL that contained or lacked apoC3, with the null hypothesis of equal coefficients. We tested the potential interactions of age (continuously), sex, race, and type 2 diabetes status with liver fat content (continuously) by including their separate interaction terms. Additionally, we conducted sensitivity analyses additionally adjusting for current medications use (antihypertensive and lipid-lowering treatment) and sedentary time (MET-min/wk).

All analyses were conducted using SAS 14 (SAS Institute Inc., Cary, NC, USA). A two-tailed *p*-value below 0.05 was considered statistically significant.

## 3. Results

Among the 5007 MESA participants, 2308 (46.1%) were men and 2699 (53.9%) were women (Table 1). The median liver fat content was similar in men (61 HU, IQR: 55–67) and women (62 HU, IQR: 56–67). In the entire sample, a total of 800 NAFLD cases were identified (16.0%). The prevalence of NAFLD did not differ by sex. Women tended to have slightly higher total apoA1, apoC3, apoA1 in HDL that contained or lacked apoC3, HDL-C, and total cholesterol levels, as well as lower physical activity level compared with men. As expected, there was a positive correlation between plasma triglycerides and apoC3 (Spearman *r* = 0.63, *p* < 0.001) 

### 3.1. Apolipoproteins and Liver Fat Content

Total apoC3 level was associated with lower liver signal intensity (*P*_trend_ < 0.0001 in Models 1 and 2), indicating higher liver fat content (Table 2). Participants in the highest quintile of total apoA1 had lower liver fat content than participants in the lowest quintile in Models 1 and 2. While apoA1 in HDL that lacked apoC3 was inversely associated with liver fat content, apoA1 in HDL that contained apoC3 was unrelated to liver fat content. Regression coefficients for apoA1 in HDL that contained or lacked apoC3 were statistically significantly different in Model 2. Associations of apoA1 in HDL that contained or lacked apoC3 each with liver fat content were not modified by age, sex, race, or type 2 diabetes status (all *P*_interaction_ > 0.05). Further adjustment for use of antihypertensive or lipid-lowering medications or sedentary time did not change our results.

### 3.2. Apolipoproteins and Non-Alcoholic Fatty Liver Disease

Higher total apoC3 levels were associated with a higher prevalence of NALFD in Model 1 (OR = 2.25 comparing Q5 to Q1; 95% CI: 1.74, 2.92; Table 3). This association was slightly attenuated in Model 2 (OR = 1.71 comparing Q5 to Q1; 95% CI: 1.32, 2.26). Higher total apoA1 was associated with a lower prevalence of NAFLD in both models. ApoA1 in HDL that lacked apoC3 related to a lower prevalence of NAFLD in Models 1 and 2, whereas no association was found for apoA1 in HDL that contained apoC3. Tests for heterogeneity of regression coefficients were not statistically significant.

## 4. Discussion

In a multi-ethnic group of US men and women, we found that higher total apoA1 was related to lower liver fat content and lower prevalence of NAFLD. The presence of apoC3 modified the association of apoA1 with liver fat content and NAFLD. Higher apoA1 in HDL that lacked apoC3 was related to lower fat content and lower prevalence of NAFLD. In contrast, apoA1 in HDL that contained apoC3 was unrelated with liver fat content and NAFLD.

Previous cross-sectional studies found an inverse association between HDL cholesterol and liver fat content and NAFLD prevalence [16,17]. In prospective studies, lower baseline HDL cholesterol levels predicted risk of NAFLD during follow-up [18,19]. In contrast to cholesterol content, the total HDL particle concentration was not associated with risk of NAFLD over 10 years of follow-up in the Young Finns Study [20]. Moreover, the results from reports focusing on different HDL features suggested that the association of HDL with liver fat content might depend on HDL particle size, reflecting functional heterogeneity of HDL [16,21]. Accumulation of free cholesterol in the liver might be associated with lower circulatory HDL levels in individuals with steatosis. There is a growing body of evidence that NAFLD results in increased synthesis of cholesterol with decreased capability to export it to peripheral tissues [22]. Thus, an increased efflux of cholesterol to apoA1 in nascent HDL might lead to less liver fat accumulation [23].

To the best of our knowledge, this is the first observational study that investigated the association of plasma HDL subspecies according to apoC3 presence with liver fat content and NAFLD. Our results of an inverse association between apoA1 and liver fat content were attenuated in the presence of apoC3 in HDL. In our prior work in MESA and three additional cohorts, we found that apoA1 in HDL that contained apoC3 was directly associated with CHD risk, whereas apoA1 in HDL that lacked apoC3 was inversely associated with CHD risk [10]. The two HDL subspecies were also divergently associated with risk of diabetes in the Danish Diet, Cancer, and Health Study [24], and MESA [9]. Mechanistically, insulin resistance, strongly linked to NAFLD, could explain the differential association of HDL subspecies with NAFLD. Supporting this mechanism, previously, only HDL lacking apoC3 was inversely associated with fasting plasma glucose, glycated hemoglobin in MESA [9]. Higher levels of circulating insulin and altered adipokine profile could suppress ABCA1, leading to the decreased assembly of HDL particles in the liver [25,26]. Cholesteryl ester transfer protein in the presence of hypertriglyceridemia mediates the transfer of triglycerides from triglyceride-rich lipoproteins to HDL [27]. HDL enriched in triglycerides has been efficiently hydrolyzed by hepatic lipase [28]. ApoC3 inhibits lipoprotein lipase and hepatic lipase and limits hepatic uptake of triglyceride-rich particles [5]. The presence of apoC3 on HDL could further enhance the catabolism of HDL particles [29]. The latter findings could be a potential explanation for no association of apoA1 in HDL that contained apoC3 and liver fat content in the current study.

There were some limitations in the current study. Because the present investigation has a cross-sectional design, we could not infer about temporality of associations, and reverse causation cannot be excluded. Additionally, our study did not include data on *APOC3* polymorphisms, which substantially influenced the levels of circulating apoC3 previously [6]. Another limitation is that we did not assess other features responsible for HDL heterogeneity like size or other apolipoprotein content. The prevalence of NAFLD in our study is lower than the estimated value for the population. However, a similar proportion was reported in other population-based cohorts like the Framingham Heart Study (15.3%) [30]. Moreover, we did not have information on the presence or severity of liver fibrosis, which reflects severity and prognosis of NAFLD. Lack of liver function biomarkers such as transaminases limits potential translation of our findings to clinical practice.

In the current study, one strength is the use of CT scans to quantify liver fat content. CT scans show greater sensitivity and specificity than ultrasonography routinely used in population-based studies. Although biopsy is a gold standard method, it cannot be applied in general population due to its invasiveness. Further, we were able to quantify liver fat content on a continuous scale [31]. Additional strengths included that we leveraged a large multiethnic population-based sample of US men and women, and a recent systematic review recognized an important difference in the prevalence and severity of NAFLD between different ethnic groups [32]. We were able to include factors previously recognized as important risk factors for NAFLD, such as smoking and physical inactivity [33,34]. Similarly, apoC3 levels are associated with lifestyle factors [35]. The use of apoA1 allowed for a reliable measurement of HDL. Moreover, a particular strength was the investigation of novel HDL subspecies according to the presence of apoC3.

## 5. Conclusions

In conclusion, apoA1 in HDL that lacked apoC3 was inversely associated with liver fat content and presence of NAFLD. In contrast, no association was found for apoA1 in HDL that contained apoC3. The study highlights the need for long-term prospective studies of apoA1 in HDL that contain or lack apoC3 and liver fat content, followed by assessing the relevance of these HDL subspecies as targets for the primary prevention of NAFLD. Moreover, as we only had CT scans available for evaluation of liver fat, our findings warrant replication in a setting with improved liver fat measures, potentially including tissue biopsies and fibrosis assessments.

## Figures and Tables

**Table 1:** Characteristics of 5007 participants with apolipoprotein and liver fat content measures in the Multi-Ethnic Study of Atherosclerosis at baseline (2000–2002).

	Mean (SD)	
Characteristics	Men	Women
*n*	2308	2699
Age, years	62.8 (10.2)	62.7 (10.3)
Postmenopausal women, *n* (%)	–	2269 (84.1)
Race/ethnicity, *n* (%)		
White	830 (36.0)	927 (34.4)
Chinese-American	320 (13.9)	341 (12.6)
African-American	656 (28.4)	811 (30.1)
Hispanic	502 (21.8)	620 (23.0)
Current smoker, *n* (%)	295 (12.8)	285 (10.6)
Current alcohol consumption ≥1 drink/wk, *n* (%)	1074 (46.5)	614 (22.8)
Income <$75,000/year, *n* (%)	1738 (75.3)	1694 (62.8)
Moderate to vigorous PA, MET-hr/wk, median (IQR)	70 (35–137)	56 (30–111)
NAFLD, *n* (%)	380 (16.5)	420 (15.6)
Liver fat content, HU^1^	61 (55–67)	62 (56–67)
Diabetes, *n* (%)	337 (14.6)	316 (11.7)
Hypertension, *n* (%)	1010 (43.7)	1216 (47.7)
Use of BP-lowering drugs, *n* (%)	856 (37.1)	1047 (38.9)
Use of lipid-lowering drugs, *n* (%)	397 (17.2)	461 (17.0)
BMI_LFA_, kg/m^2^	28.4 (3.9)	28.3 (5.5)
Waist circumference, cm	99 (12.1)	97.6 (15.8)
Systolic BP, mmHg	126 (19)	128 (23)
Biomarkers, mg/dL, median (IQR)		
HDL cholesterol	43 (37–51)	53 (45–64)
ApoA1	116 (99–133)	135 (118–158)
ApoA1 in HDL that contained apoC3	7.1 (5.6–8.7)	8.7 (1.0–11.0)
ApoA1 in HDL that lacked apoC3	111 (93–124)	127 (110–148)
Proportion of HDL that contained apoC3, %	6.1 (5.3–7.1)	6.5 (5.5–7.4)
ApoC3	8.2 (6.4–10.4)	9.1 (7.3–11.4)
Triglycerides	113 (79–166)	109 (78–158)
Total cholesterol	185 (164–208)	197 (176–220)

ApoA1, apolipoprotein A1; ApoC3, apolipoprotein C3; BMI_LFA_, liver-fat-adjusted body mass index; BP, blood pressure; HDL, high-density lipoprotein; IQR, interquartile range; MET, metabolic equivalent; NAFLD, non-alcoholic fatty liver disease; PA, physical activity.

**Table 2:** Least-square means of baseline liver fat content by apolipoprotein quintile in MESA.

	Liver Fat Content (95% CI), HU (Lower HU Indicate Higher Liver Fat Content)					
	Q1	Q2	Q3	Q4	Q5	*P*_trend_
**Total apoC3**						
Median levels, mg/dL	5.4	7.3	8.6	10.4	13.9	
Model 1	61.1 (60.4, 61.8)	60.5 (59.8, 61.1)	60.1 (59.4, 60.7)	58.8 (58.1, 59.4)	57.1 (56.5, 57.8)	<0.0001
Model 2	61.0 (60.2, 61.7)	60.3 (59.5, 61.1)	59.9 (59.1, 60.7)	58.6 (57.8, 59.4)	57.0 (56.2, 57.8)	<0.0001
**Total apoA1**						
Median levels, mg/dL	91.8	111.7	125.8	142.4	174.6	
Model 1	57.9 (57.2, 58.6)	59.0 (58.4, 59.7)	59.8 (59.1, 60.5)	59.8 (59.1, 60.5)	61.2 (60.5, 61.9)	<0.0001
Model 2	58.0 (57.2, 58.8)	59.1 (58.3, 59.9)	59.7 (58.9, 60.4)	59.7 (58.9, 60.5)	60.9 (60.0, 61.7)	<0.0001
**ApoA1 in HDL that lacked apoC3**						
Median levels, mg/dL	85.9	104.7	117.7	132.8	163.2	
Model 1	58.0 (57.3, 58.7)	59.1 (58.4, 59.8)	59.8 (59.1, 60.5)	59.9 (59.2, 60.6)	61.0 (60.2, 61.7)	<0.0001
Model 2	58.1 (57.2, 58.9)	59.2 (58.4, 60.0)	59.7 (58.9, 60.5)	59.7 (58.9, 60.5)	60.6 (59.7, 61.5)	<0.0001
**ApoA1 in HDL that contained apoC3**						
Median levels, mg/dL	5.0	6.6	7.9	9.3	12.3	
Model 1	59.1 (58.4, 59.9)	59.1 (58.4, 59.8)	59.4 (58.7, 60.1)	60.1 (59.4, 60.8)	60.0 (59.2, 60.8)	0.71
Model 2	59.4 (58.5, 60.2)	59.2 (58.4, 60.0)	59.2 (58.4, 60.0)	59.8 (59.0, 60.6)	59.8 (58.9, 60.6)	0.57

ApoA1 in HDL that contained or lacked apoC3 was modeled simultaneously; HU, Hounsfield units. Model 1 was adjusted for age, sex, race/ethnicity (White/African-American/Chinese-American/Hispanic) and study site. Model 2 included covariates from Model 1 and was further adjusted for smoking status (current/former/never), alcohol status (current/former/never) and intake (g/day), income (four categories), liver fat-adjusted BMI (kg/m^2^), and physical activity (MET-hr/week). Test for heterogeneity of regression slopes for apoA1 in HDL that contained or lacked apoC3: Model 1: *P* = 0.003, Model 2: *P* = 0.048.

**Table 3:** Odds ratios for non-alcoholic fatty liver disease by apolipoprotein quintile in MESA.

	Odds Ratios for Non-Alcoholic Fatty Liver Disease (95% CI)						
	Q1	Q2	Q3	Q4	Q5	Per 1-SD	*P*_trend_
**Total apoC3**							
*n* of cases	112	127	146	178	237	800	
Model 1	1.0 (ref.)	1.17 (0.88, 1.54)	1.34 (1.02, 1.76)	1.70 (1.30, 2.22)	2.25 (1.74, 2.92)	1.27 (1.18, 1.36)	<0.0001
Model 2	1.0 (ref.)	1.19 (0.90, 1.57)	1.36 (1.03. 1.78)	1.73 (1.32, 2.26)	1.73 (1.32, 2.26)	1.27 (1.18, 1.36)	<0.0001
**Total apoA1**							
*n* of cases	203	187	152	150	108	800	
Model 1	1.0 (ref.)	0.92 (0.73, 1.16)	0.73 (0.57, 0.93)	0.73 (0.57, 0.94)	0.49 (0.37, 0.64)	0.79 (0.72, 0.86)	<0.0001
Model 2	1.0 (ref.)	0.90 (0.72, 1.14)	0.72 (0.56, 0.92)	0.71 (0.55, 0.91)	0.47 (0.35, 0.62)	0.77 (0.71, 0.85)	<0.0001
**ApoA1 in HDL that lacked apoC3**							
*n* of cases	192	191	155	139	123	800	
Model 1	1.0 (ref.)	0.88 (0.70, 1.12)	0.75 (0.58, 0.97)	0.76 (0.57, 1.00)	0.56 (0.41, 0.77)	0.81 (0.73, 0.91)	0.0002
Model 2	1.0 (ref.)	0.86 (0.68, 1.10)	0.73 (0.56, 0.94)	0.74 (0.56, 0.98)	0.53 (0.39, 0.74)	0.80 (0.72, 0.89)	<0.0001
**ApoA1 in HDL that contained apoC3**							
*n* of cases	207	181	155	146	111	800	
Model 1	1.0 (ref.)	1.08 (0.85, 1.37)	0.94 (0.72, 1.22)	0.88 (0.67, 1.17)	0.85 (0.62, 1.16)	0.95 (0.85, 1.06)	0.34
Model 2	1.0 (ref.)	1.10 (0.86, 1.40)	0.95 (0.73, 1.23)	0.89 (0.67, 1.18)	0.85 (0.62, 1.17)	0.95 (0.85, 1.05)	0.31

ApoA1 in HDL that contained or lacked apoC3 was modeled simultaneously. Model 1 was adjusted for age, sex, race/ethnicity (White/African-American/Chinese-American/Hispanic) and study site. Model 2 included covariates from Model 1 and was further adjusted for smoking status (current/former/never), alcohol status (current/former/never) and intake (g/day), income (four categories), liver fat-adjusted BMI (kg/m^2^), and physical activity (MET-hr/week). Test for heterogeneity of regression slopes for apoA1 in HDL that contained or lacked apoC3: Model 1: *P* = 0.10, Model 2: *P* = 0.09.
